# Outcomes When Using Adjunct Dexmedetomidine with Propofol Sedation in Mechanically Ventilated Surgical Intensive Care Patients

**DOI:** 10.3390/pharmacy6030093

**Published:** 2018-08-28

**Authors:** Jessica M. Louie, Nick W. Lonardo, Mary C. Mone, Vanessa W. Stevens, Rishi Deka, Wayne Shipley, Richard G. Barton

**Affiliations:** 1 Department of Pharmacy, University of Utah Health, Salt Lake City, UT 84132, USA; nick.Lonardo@hsc.utah.edu (N.W.L.); wayne.Shipley@hsc.utah.edu (W.S.); 2 Department of Pharmacy Practice, West Coast University School of Pharmacy, Los Angeles, CA 90004, USA; 3 Department of Surgery, University of Utah Health, Salt Lake City, UT 84132, USA; Mary.Mone@hsc.utah.edu (M.C.M.); Richard.Barton@hsc.utah.edu (R.G.B.); 4 Department of Internal Medicine, University of Utah School of Medicine, Salt Lake City, UT 84132, USA; Vanessa.Stevens@pharm.utah.edu; 5 Department of Pharmacy Practice, University of Utah College of Pharmacy, Salt Lake City, UT 84132, USA; rishideka@gmail.com

**Keywords:** sedation agents, delirium, adjunctive use, comparative effective research, intensive care length of stay

## Abstract

**Objective:** Compare the duration of mechanical ventilation between patients receiving sedation with continuous infusions of propofol alone or combination with the use of dexmedetomidine and propofol. **Design:** Retrospective, propensity matched (1:1) cohort study, employing eight variables chosen a priori for matching. Timing of exposure to dexmedetomidine initiation was incorporated into a matching algorithm. **Setting:** Level 1, university-based, 32-bed, adult, mixed trauma and surgical intensive care unit (SICU). Continuous sedation was delivered according to a protocol methodology with daily sedation vacation and spontaneous breathing trials. Choice of sedation agent was physician directed. **Patients:** Between 2010 and 2014, 149 SICU patients receiving mechanical ventilation for >24 h received dexmedetomidine with propofol. Propensity matching resulted in 143 pair cohorts. **Interventions:** Dexmedetomidine with propofol or propofol alone. **Measurements and Main Results:** There was no statistical difference in SICU length of stay (LOS), with a median absolute difference of 5.3 h for propofol alone group (*p* = 0.43). The SICU mortality was not statistically different (RR = 1.002, *p* = 0.88). Examining a 14-day period post-treatment with dexmedetomidine, on any given day (excluding days 1 and 14), dexmedetomidine with propofol-treated patients had a 0.5% to 22.5% greater likelihood of being delirious (CAM-ICU positive). In addition, dexmedetomidine with propofol-treated patients had a 4.5% to 18.8% higher likelihood of being above the target sedation score (more agitated) compared to propofol-alone patients. **Conclusions:** In this propensity matched cohort study, adjunct use of dexmedetomidine to propofol did not show a statistically significant reduction with respect to mechanical ventilation (MV) duration, SICU LOS, or SICU mortality, despite a trend toward receiving fewer hours of propofol. There was no evidence that dexmedetomidine with propofol improved sedation scores or reduced delirium.

## 1. Introduction

Mechanical ventilation is a life-saving intervention utilized in 20–30% of intensive care unit (ICU) admissions, but it is associated with many risks. The use of mechanical ventilation is one of the costliest interventions in the ICU, accounting for an estimated $27 billion, or 12% of all hospital costs [1,2,3]. While receiving mechanical ventilation, the delivery of sedation and analgesic agents are often necessary supportive treatments which are highly effective in providing comfort, tolerance, and improving ventilator synchrony [2]. However, there are known consequences to using sedative medications, including potentially prolonged duration of mechanical ventilation, increased overall ICU length of stay (LOS), and delirium [4,5,6].

One of the major goals for critical care clinicians is achieving a balance between adequate levels of sedation without causing a concomitant prolonged need for ventilator support. In the age of evidence-based medicine, clinicians refer to society-specific guidelines for treatment direction. The 2013 Society of Critical Care Medicine Pain, Agitation, and Delirium guidelines recommend using a non-benzodiazepine sedative, propofol or dexmedetomidine, to improve clinical outcomes, but the recommendations do not address the adjunctive use of agents, such as the combined use of dexmedetomidine with propofol [2].

Dexmedetomidine presents an attractive advantage to other sedative agents because it does not cause respiratory depression, which allows for the patient to be extubated while continuing the sedative [7]. Initially marketed for short-term use (<24 h), dexmedetomidine is now being used as a primary sedative as well as an adjunct agent to propofol or a benzodiazepine [5,7,8,9,10,11,12,13,14,15,16]. Despite randomized controlled trials (RCT) showing that dexmedetomidine is associated with a shorter duration of mechanical ventilation compared to benzodiazepines, the “propofol compared to dexmedetomidine” (termed PRODEX) trial, which compared the exclusive use of either dexmedetomidine or propofol, failed to show a statistical difference [5,13,15]. Other studies have shown that adjunctive dexmedetomidine may reduce opiate and sedative use and facilitate extubation, but most of these lacked control groups [8,9,10,11,12,13]. Notably, there is evidence that the majority of dexmedetomidine use is adjunctive rather than exclusive when the indication is to sedate mechanically ventilated patients [17,18,19]. Because of this variation in treatment use with dexmedetomidine in the literature, we chose to examine our institution’s use of this treatment. The goal of this study was to evaluate a mixed trauma and surgical cohort of mechanically ventilated ICU patients, who require ventilation more than 24 h, in order to determine if the unrestricted addition of dexmedetomidine to patients already receiving propofol would result in shorter mechanical ventilation duration or other improved outcomes.

## 2. Materials and Methods

**Study Population**

Patient data was collected at a Level 1 trauma university hospital, within a 32-bed mixed trauma and surgical intensive care unit (SICU). Critical care was provided by a combined group of general and trauma surgeons, emergency room physicians, and anesthesiologists. The SICU employs protocol-driven sedation regimens directing dosage and titration, with daily sedation vacations and spontaneous breathing trials. The use of dexmedetomidine is not restricted by formulary control and physicians may use it without regard to specific regulations.

A query in July 2014 identified adult patients from March 2010 through May 2014 requiring mechanical ventilation. This study was approved by the Institutional Review Board and met federal exemption status.

Mechanical ventilation was defined as any form of ventilator support, including continuous positive airway pressure. Study inclusion required the use of mechanical ventilation for >24 h and continuous infusion of dexmedetomidine with propofol or with propofol alone. For patients receiving mechanical ventilation support for more than a single event (or intubation event) during their SICU stay, only the first event that was >24 h in length was analyzed.

**Figure 1:**
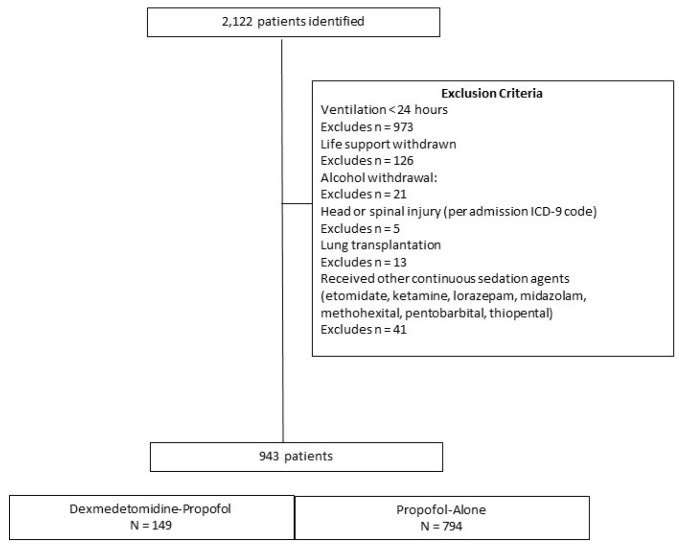
Flow diagram of enrollment with exclusion criteria and group totals.

Study exclusions included: age < 18 years, life support withdrawn, alcohol withdrawal, severe head injuries, cervical spine fractures with cord involvement, lung transplantation, patients receiving dexmedetomidine as a single agent, or treatment with a continuous infusion of etomidate, ketamine, lorazepam, midazolam, or a barbiturate (Figure 1 and Supplement).

**Outcome Measures**

The primary outcome was mechanical ventilation duration. Secondary outcomes included SICU LOS and all-cause SICU mortality. Exploratory outcomes included: the daily prevalence of delirium, achievement of target Riker Sedation-Agitation Scale scores, bradycardia (heart rate (HR) < 50 beats), hypotension (mean arterial pressure (MAP) < 60 mmHg), concomitant antipsychotic medication use, tracheostomy, and triglyceride level > 200 mg/L.

**Statistical Methods**

We conducted a retrospective, propensity-matched, cohort study evaluating the treatment effect of adding dexmedetomidine to propofol. To balance the treatment cohorts with similar variables in the absence of randomization, we used propensity scoring to match the probability of receiving propofol alone versus receiving propofol plus adjunctive dexmedetomidine [20]. Candidate variables for the propensity score model were identified a priori to any analyses being performed and included variables thought to be true confounders [21,22]. Variables strongly related to the outcome but with a questionable relationship to the treatment (risk factors) were included. Those variables definitely related to the treatment but only possibly related to the outcome were excluded [23]. The eight variables chosen were age, SICU admission Acute Physiology and Chronic Health Evaluation (APACHE) II score, gender, chronic obstructive pulmonary disease (COPD), hemodynamic instability, admitting service, surgical status, and calendar time of treatment (Supplement).

All patients had initiated treatment with propofol at the time of mechanical ventilation, and dexmedetomidine may have been initiated at any time during mechanical ventilation. A time-dependent bias arises when a time-varying exposure is treated as if it were present at baseline, and results in inflating the impact of the drug on outcomes [24]. In order to account for the time-varying nature of the exposure, time to dexmedetomidine start was incorporated into the matching algorithm. For each patient that initiated dexmedetomidine at time *t* hours, the pool of eligible controls consisted of all patients who were currently receiving only propofol at time *t.* Within the subgroup of eligible controls, patients were then matched on the propensity score using a greedy matching algorithm and 0.2 standard deviation caliper width. Comparability between treatment groups was assessed using standardized differences and distributions of propensity scores before and after matching.

**Figure 2:**
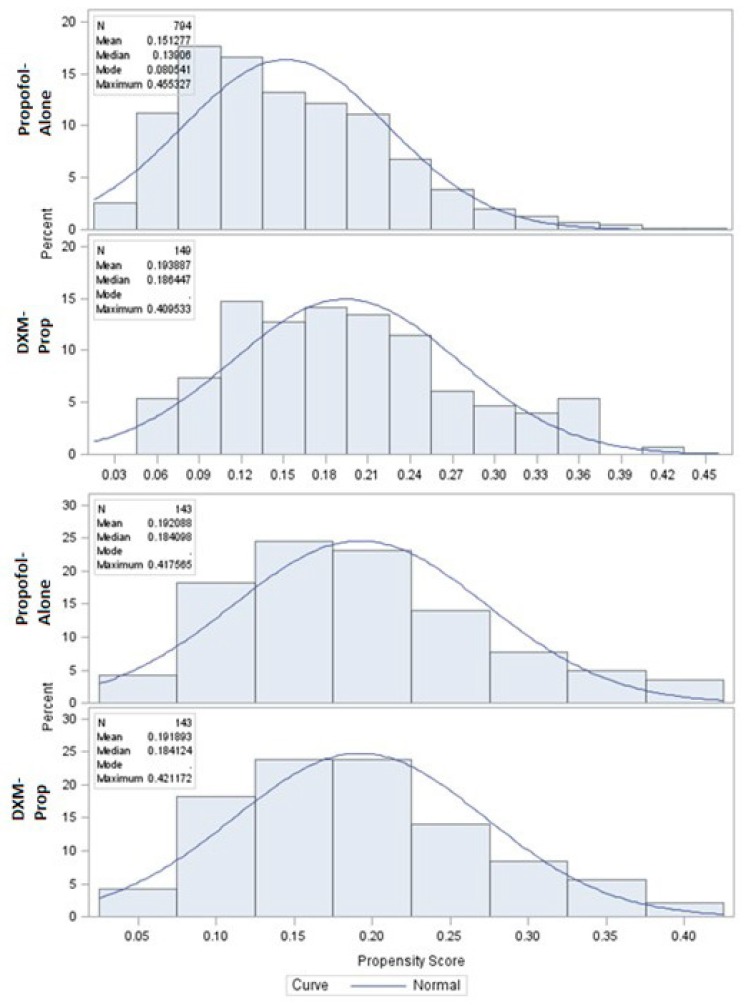
Propensity score distribution plot comparing unmatched and matched scores between dexmedetomidine-propofol and propofol-alone treated patients.

**Figure 3:**
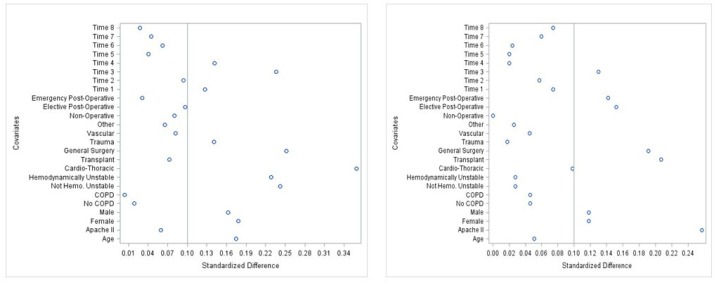
Scatter plots illustrating the standard difference of 8 covariates before and after matching. Scatter plots illustrating the standard difference of 8 covariates before matching (*left*) and after matching (*right*). A significant narrowing of the standard difference demonstrates successful propensity score matching between the sedation groups. Definition of abbreviations: APACHE = Acute Physiology and Chronic Health Evaluation; COPD = Chronic Obstructive Pulmonary Disease.

Baseline demographic and clinical characteristics were summarized using medians, interquartile ranges (IQR), frequencies, and percentages. The effect of adding dexmedetomidine to propofol sedation was assessed in the matched cohort using regression models. Models included the treatment group as the primary independent variable and APACHE II, due to a minor residual imbalance between groups, in the matched sample. Mechanical ventilation duration and SICU LOS were modeled using generalized linear models (GLM). Modified Park tests indicated that the distributions for mechanical ventilation duration and SICU LOS were Poisson and Gaussian, respectively. Due to the presence of over-dispersion and heteroscedasticity, mechanical ventilation duration was modeled as a function of treatment group and APACHE II score using a negative binomial model with a log link. The treatment group effect on SICU LOS was estimated using a GLM regression with a Gaussian distribution and log link adjusted for APACHE II score. Log binomial regression was used to estimate the impact of dexmedetomidine on SICU mortality after accounting for the impact of APACHE II score. Figure 2 and Figure 3 illustrate the successful narrowing of standardized differences of variables after propensity score matching.

Exploratory outcomes were subjected to univariate analysis using Wilcoxon rank sum tests and Chi-square and Fisher’s exact tests. Statistical analyses were performed in SAS V9.3 (Cary, NC, USA) and Stata 13 (College Station, TX, USA), assuming a two-sided alpha of 0.05.

## 3. Results

A total of 943 admissions met the inclusion criteria for the sedation groups (Figure 1). Treatment with propofol alone was most common, 84.1% (n = 794) compared to dexmedetomidine-propofol (n = 149). After 1:1 matching, there were 143 matched pairs (Table 1). The only statistically different variable after matching was admission APACHE II score, median score of 16 (interquartile range (IQR) 6.0) in the dexmedetomidine-propofol group versus 17 (IQR 8.0) in the propofol alone group, *p* = 0.03.

Crude results for mechanical ventilation duration showed no statistical difference in the mean or median between treatment groups before adjusting for APACHE II score. After adjusting for APACHE II, there was still no difference between the dexmedetomidine-propofol (137.0 h) and propofol alone groups (142.8 h) (risk ratio (RR) = 1.086, 95% confidence interval (CI) 0.924–1.275, *p* = 0.31) (Table 2).

Crude results for the difference in SICU LOS showed no statistical difference in the mean or median number of hours between treatment groups before or after adjusting for APACHE II score. There was no difference between the dexmedetomidine-propofol (217.9 h) and propofol alone groups (212.6 h) (RR = 0.937, 95% CI 0.799–1.100, *p* = 0.43) (Table 2).

There was no statistical difference in all-cause SICU mortality between the dexmedetomidine-propofol and propofol alone groups (RR = 1.002, 95% CI 0.967–1.038, *p* = 0.88) (Table 2).

**Figure 4:**
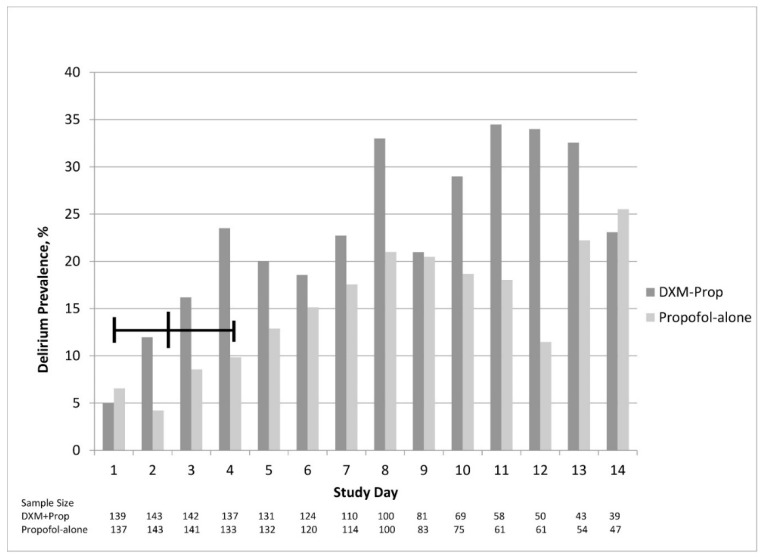
Daily Prevalence of Delirium through day 14 or Discharge from ICU in Matched Cohort. Delirium diagnosed using the Confusion Assessment Method for the Intensive Care Unit (CAM-ICU). On any given day (excluding study day 1 & 14), DXM-Prop treated patients had a 0.5% to 22.5% greater likelihood of being delirious (CAM-ICU positive) compared to propofol alone. Bold vertical arrow indicates median time (days) from propofol initiation to start of DXM after intubation. Definition of abbreviations: DXM = dexmedetomidine.

**Figure 5:**
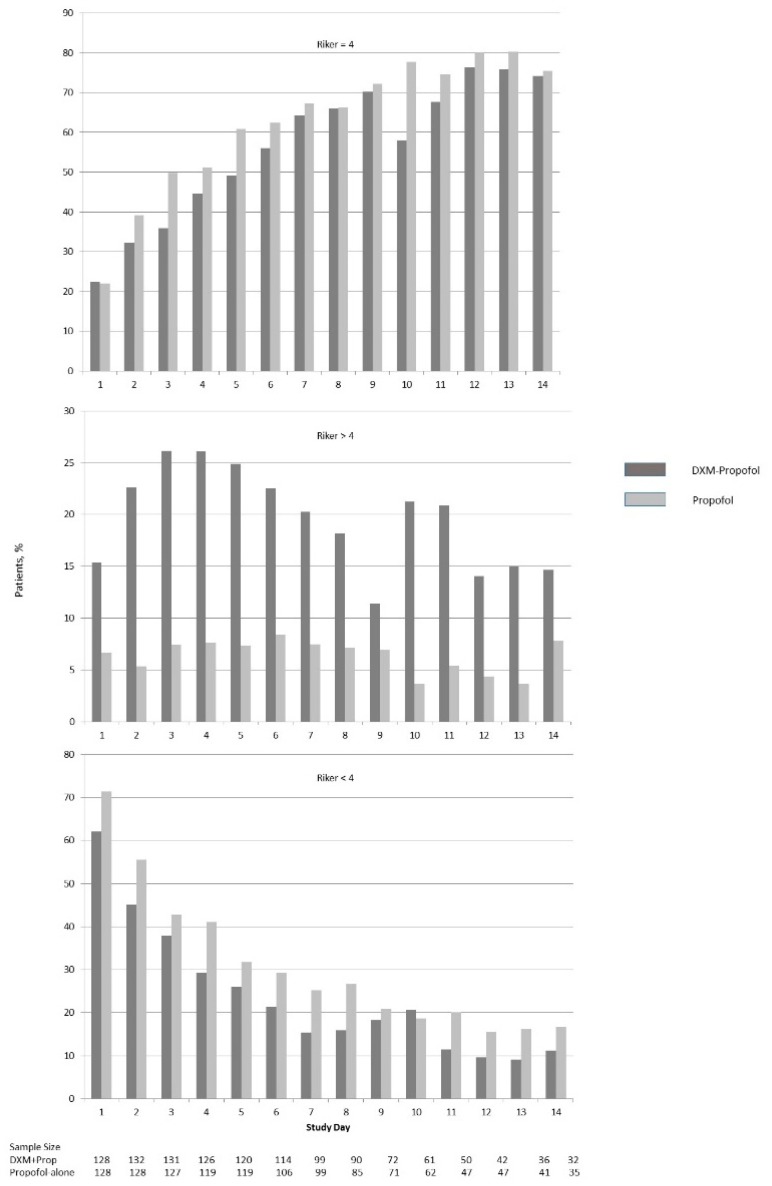
Percentage of Patients with Riker Sedation Score of at goal, above goal or below goal through day 14 or Discharge from ICU in Matched Cohort. (a) Percentage of patients meeting target goal on Riker Sedation-Agitation Scale of 4 while receiving sedative medication. On any given day (excluding study day 1), DXM-Prop treated patients had a 0.3% to 19.6% lower likelihood of being at target sedation score compared to those treated with propofol alone. (b) Percentage of patients who were above target goal on Riker Sedation-Agitation Scale (Riker > 4) while receiving sedative medication. On any given day, DXM-treated patients had a 4.5% to 18.8% higher likelihood of being above target sedation score (more agitated) compared to propofol-alone treated patients. (c) Percentage of patients who were below target goal on Riker Sedation-Agitation Scale (Riker < 4) while receiving sedative medication. On any given day (excluding study day 10), DXM-treated patients had a 2.5% to 11.8% lower likelihood of being below target sedation score (more sedated) compared to propofol-alone treated patients.

Table 3 displays the results for continuous exploratory variables. Doses were calculated based on the total volume fluid administered (milliliters (mL)), documented dosing weight, and order duration. The propofol infusion duration was lower in the dexmedetomidine-propofol group, with an absolute median difference of 22.6 h, *p* = 0.07. Figure 4 and Figure 5 illustrate the differences in delirium and sedation scores during each study day. Table 4 and Table 5 display results for categorical exploratory variables. There were statistically significant differences in rates of hypotension (76.2% versus 53.8%, *p* < 0.001), bradycardia (5.6% versus 19.6%, *p* < 0.001), and antipsychotic medication use (68.5% versus 52.4%, *p* = 0.005) for the dexmedetomidine-propofol and propofol alone groups, respectively.

## 4. Discussion

In this analysis of SICU patients, we found that the use of dexmedetomidine as an adjunct sedative in patients already receiving propofol provided no obvious benefit in terms of mechanical ventilation duration, time in the SICU, and all-cause SICU mortality when compared to a propensity matched group of patients receiving propofol alone. In addition, the prevalence of delirium did not appear to decrease after dexmedetomidine was added to propofol-based sedation.

Mechanical ventilation is one of the most important treatments delivered in the critical care setting, but as an invasive intervention, there are known associated risks. The risks are directly associated with the placement and maintenance of the endotracheal tube, the function of the ventilator with pressure and oxygen delivery, and infections such ventilator-associated pneumonia (VAP), as well as indirectly associated with cases involving prolonged immobility, and increased risk for stress-related gastrointestinal ulceration. As the duration of mechanical ventilation increases, these risks increase correspondingly [25,26,27]. Because of the relationship between sedation and mechanical ventilation duration, there is a strong focus among critical care scholars to provide guidelines regarding proper sedative selection as well as optimal sedation levels [4].

In 2006, Dasta et al. undertook a retrospective analysis of propofol-midazolam versus dexmedetomidine-propofol-midazolam [17]. They found that dexmedetomidine was used as a single agent in only 5% of patients. In 2014, we examined a multicenter intensive care database of patients ventilated for more than 48 h that used a single sedative, and found that dexmedetomidine was rarely used exclusively (0.6% of 13,770 cases) [18]. Both of these studies underscore the infrequent use of dexmedetomidine as an exclusive sedative and consequently should give pause to accepting the application of study results in which dexmedetomidine was investigated as a single sedative agent [5,15,16,17,18,19,28].

The literature for dexmedetomidine use can be summarized by a published RCT that used dexmedetomidine as an exclusive sedative, or as an adjunctive sedative added to either propofol or a benzodiazepine. In the context of comparing and contrasting our results, we focused only on the adjunctive use of dexmedetomidine [5,16,28]. Between 2003 and 2016, eight studies investigated dexmedetomidine as an adjunctive sedative (Table 6) [8,9,10,11,12,13,14,17]. Six of these were observational studies and all suggested some benefit to dexmedetomidine, but none of these studies had a comparison cohort [8,9,10,11,12,13]. Dasta et al. described outcomes suggesting benefit when dexmedetomidine was used with midazolam and propofol [17]. However, this study did not balance the cohorts and combined the propofol cohort with midazolam. Given that multiple studies have shown that benzodiazepines have worse outcomes when compared to propofol sedation, the associations reported are not relevant to the results we present [2,11,16,17,18,29]. In a recent RCT of the adjunctive use of dexmedetomidine, Reade et al. examined data from a mixed ICU in ventilated patients with severe agitated delirium (hyperactive delirium) [14]. The dexmedetomidine cohort had a statistically significant increase in ventilator-free hours and earlier extubation. In addition, dexmedetomidine patients had statistically less delirium. While these results are encouraging, it is important to note that hyperactive delirium is less common than hypoactive delirium, 20% vs. 80%, and purely hyperactive delirium accounts for <5% of cases in the ICU population [2,30]. In addition, only 74 of 21,500 patients screened met the inclusion criteria, limiting the generalizability of the findings. Although it would seem that these results challenge the outcomes of our study, it may merely underscore that using adjunctive dexmedetomidine with propofol in a non-selective patient population (i.e., delirious, but not hyperactive) may not deliver the same beneficial outcomes.

One of the principle reasons for using dexmedetomidine adjunctively is to reduce the use of other more deliriogenic sedative agents. We found that those patients treated with both dexmedetomidine and propofol had fewer hours of propofol infused, and although this was not statistically significant, it was nearly 24 h less (*p* = 0.07) (Table 3). This reduction in propofol did not appear to translate into a positive effect on ventilator time or duration in the SICU. Although we expected the rate of delirium and use of antipsychotic medications to be higher in the dexmedetomidine-propofol cohort due to the inherent treatment bias for patients not responding to propofol, we anticipated that the addition of dexmedetomidine might improve the delirium and sedation scores. This did not occur, despite a trend toward less propofol use. The use of antipsychotic medications was greater among patients treated with dexmedetomidine-propofol (Table 3, Table 4 and Table 5). Because this study was not designed to show causality, the reported exploratory outcomes should be interpreted cautiously. It is notable, however, that the daily prevalence of delirium did not appear to decrease after dexmedetomidine was added, which is commonly a cited reason why dexmedetomidine is used (Figure 4). Moreover, a larger percentage of patients in the dexmedetomidine-propofol group continued to have Riker scores > 4 throughout their ICU stay, while patients in the propofol alone group had more Riker scores < 4 throughout their ICU stay (Figure 5).

In 2007, MacLaren et al. published a retrospective study evaluating dexmedetomidine adjunctively added to propofol, midazolam, or lorazepam [11]. Riker scores were at goal (3–4) more often in the 24 h before adding dexmedetomidine compared to after. In the 24-h periods before and after adding dexmedetomidine, severe agitation occurred more frequently after dexmedetomidine was added to other sedatives (30% versus 10%, *p* = 0.05). These pre- and post-addition dexmedetomidine results are qualitatively similar to those reported in our study (Figure 4).

Our exploratory outcomes evaluated cardiovascular effects related to sedative use. Our results demonstrated statistical differences for hypotension and bradycardia (Table 4). The hypotension findings were not surprising since both sedatives are known for this side effect. The reported bradycardia rate for dexmedetomidine is 5%–7%, and in this analysis was 5.6% [7]. Our bradycardia rate in the propofol alone group was 19.6% compared with a package insert rate of 1%–3% [31]. Possible explanations may be related to the high percentage of cardiothoracic patients (45%–50%) in the matched study cohort, who often experience bradycardia after cardiac valve surgery.

The sedative used for individual patients in this unit is physician-directed, combined with protocols for standardized sedation goals which actively involve weaning one agent as another is added (Supplement). Our rationale for examining this data was based on the increased presence, albeit slow in progression, of adjunctive dexmedetomidine use within the unit, which, prior to this, was exclusively propofol-based. In order to ascertain if this introduction of a more expensive agent in combination with the standard agent was advantageous or not, we retrospectively examined the evidence available to us.

There are strengths to this observational cohort study. To our knowledge, this is the only matched propensity score study that examined ventilation duration when dexmedetomidine was added to a propofol-based sedation regimen. In addition, our study setting is free of continuous infusion benzodiazepines, which are discouraged by the 2013 sedation guidelines [2]. The study cohorts were matched on confounders known to be predictors related to ventilation duration, SICU LOS, and mortality. Additionally, the propensity score matching included time intervals to account for possible practice changes by intensivists, guidelines, or unit protocols. Most importantly, the time-varying exposure of dexmedetomidine was incorporated into the matching and allowed us to reduce selection bias by removing a concern that a portion of the propofol-alone patients would be extubated so rapidly that they would not have an opportunity to receive dexmedetomidine. We also quantified relevant exploratory outcomes to help explain the treatment effect of adding dexmedetomidine to a propofol-based sedation regimen.

There are several limitations to this retrospective observational study that should be discussed. First, we could not match for delirium or agitation in the propensity model. This is because there is no specific counterfactual patient match in the propofol alone group due to the random variation of dexmedetomidine start times and the absence of a fixed stationary time in which to match for delirium. Second, there was likely a selection bias for patients that were more difficult to sedate in the dexmedetomidine-propofol cohort, which is evident by the higher prevalence of delirium and Riker scores > 4 in the days preceding the median time of starting dexmedetomidine (Figure 4 and Figure 5). This may in part explain why we did not detect a significant reduction in the duration of mechanical ventilation. By including both delirious and non-delirious patients in the same analysis, we may have attenuated a positive effect of adding dexmedetomidine to the propofol sedation. We chose not to study selective subgroups, such as those with severe agitation, as we wanted the results to represent the generalized use of dexmedetomidine in a natural post-surgical setting. It is possible that certain subgroups may have benefited from adding dexmedetomidine and were not revealed in this study. With these limitations acknowledged, it is worth noting that although the prevalence of delirium and Riker scores were documented for a full 14 days, there was no evidence of a meaningful improvement in the dexmedetomidine-propofol cohort (Figure 4 and Figure 5). Third, as with all observational studies, there is a possibility of unmeasured covariates not accounted for in the propensity model. Fourth, as a single center study performed in a high-acuity center of trauma and surgical ICU patients, the practice pattern may not be applicable to lower acuity patients, or to those patients requiring short-term ventilation. Given these limitations, single center studies can be the necessary catalyst for undertaking an adequately powered RCT or multi-center review. Fifth, the ability to accurately assess the dose of sedative agents was difficult, secondary to the varying rates of continuous infusions. Finally, the rate of reported CAM-ICU unable-to-assess (UTA) ranged from 36%–64% each day. Acceptable rates of UTA CAM-ICU assessments have not been well defined, but a 2015 study found that 30% of UTA documents were inappropriate [32].

## 5. Conclusions

In this single-center, retrospective cohort study using propensity score matching, adding a continuous infusion of dexmedetomidine to those patients already being treated with a continuous infusion of propofol in a mixed trauma and surgical population did not decrease the mechanical ventilation duration or the overall SICU length of stay or reduce the SICU mortality rate. In addition, there was no evidence that starting dexmedetomidine reduced the prevalence of delirium. These results suggest that unrestricted use of dexmedetomidine as an adjunctive sedative to a propofol-based sedation regimen may not have the desired effect on outcomes that intensive care clinicians expect, particularly in patients who require more than 24 h of ventilator use. These results should be interpreted cautiously since there may be subgroups of patients not identified that may benefit from the adjunctive use of dexmedetomidine. A large randomized prospective trial using adjunctive dexmedetomidine is be needed to confirm or refute these results.

## Figures and Tables

**Table 1:** Unmatched and Matched Cohorts Based on Sedation Treatment Groups.

	Unmatched Cohort			Matched Cohort		
Variable	DXM-Propofol (*n* = 149)	Propofol (*n* = 794)	*p*-Value	DXM-Propofol (*n* = 143)	Propofol (*n* = 143)	*p*-Value
	(SD or IQR, N (%))			(SD or IQR, N (%))		
Age (mean)	53.5 (17.4)	56.5 (17.2)	0.05 *^a^*	53.6 (17.4)	52.7 (16.9)	0.67 *^a^*
APACHE II Score (median)	16.0 (6.0)	16.0 (9.0)	0.74 *^a^*	16.0 (6.0)	17.0 (8.0)	0.03 *^a^*
Female	45 (30.2)	305 (38.4)	0.06 *^b^*	45 (31.5)	53 (37.1)	0.32 *^b^*
Male	104 (69.8)	489 (61.6)		98 (68.5)	90 (62.9)	
COPD Diagnosis *^c^*						
No	135 (90.6)	719 (90.6)	0.98 *^b^*	129 (90.2)	127 (88.8)	0.70 *^b^*
Yes	14 (9.4)	75 (9.4)		14 (9.8)	16 (11.2)	
Hemodynamic Instability *^c^*						
No	71 (47.6)	468 (59.3)	0.008 *^b^*	74 (51.8)	71 (49.7)	0.81 *^b^*
Yes	78 (52.4)	318 (40.7)		69 (48.2)	72 (50.4)	
Admitting Service						
Cardio-Thoracic	77 (51.7)	269 (34.3)	0.001 *^b^*	72 (50.4)	65 (45.4)	0.35 *^b^*
Transplant	3 (2.0)	25 (3.2)		3 (2.1)	0 (0.0)	
General urgery	24 (16.1)	208 (26.5)		24 (16.8)	35 (24.5)	
Trauma	29 (19.5)	200 (25.5)		29 (20.3)	28 (19.6)	
Vascular	4 (2.7)	33 (4.2)		4 (2.8)	3 (2.1)	
Other	12 (8.0)	50 (6.4)		11 (7.7)	12 (8.4)	
Admission Type						
Non-Operative	50 (33.6)	229 (28.8)	0.48 *^b^*	49 (34.3)	39 (27.3)	0.37 *^b^*
Elective Postoperative	58 (38.9)	319 (40.2)		54 (37.8)	64 (44.8)	
Emergency Postoperative	41 (27.5)	246 (30.9)		40 (27.9)	40 (27.9)	
Time Period *^c^*						
1	14 (9.4)	106 (13.4)	0.13 *^b^*	14 (9.8)	11 (7.6)	0.94 *^b^*
2	24 (16.1)	101 (12.7)		22 (15.4)	25 (17.5)	
3	9 (6.0)	102 (12.8)		9 (6.3)	5 (3.505)	
4	25 (16.8)	93 (11.7)		22 (15.4)	21 (14.7)	
5	20 (13.4)	95 (11.9)		20 (13.9)	19 (13.3)	
6	14 (9.4)	89 (11.2)		14 (9.8)	13 (9.1)	
7	19 (12.8)	89 (11.2)		19 (13.3)	22 (15.4)	
8	24 (16.1)	119 (14.9)		23 (16.1)	27 (18.9)	

DXM = dexmedetomidine; APACHE = Acute Physiology and Chronic Health Evaluation; SD = standard deviation; IQR = interquartile range; COPD = Chronic Obstructive Pulmonary Disease; SICU = Surgical Intensive Care Unit; *^a^* T-Test; *^b^* Chi-Squared Test; *^c^* Variables defined in online supplement.

**Table 2:** Outcome Measures in Matched Cohort.

Outcome	DXM-Propofol (*n* = 143)	Propofol (*n* = 143)	Risk Ratio	95% Confidence Intervals	*p*-Value
Mechanical ventilation duration hours; Median (IQR)	137.0 (132.3)	142.8 (153.4)	1.086	(0.924, 1.275)	0.31
SICU length of stay hours; Median (IQR)	217.9 (178.9)	212.6 (225.6)	0.937	(0.799, 1.100)	0.43
SICU mortality; N (%)	5 (3.5)	3 (2.1)	1.002	(0.967, 1.038)	0.88

DXM = dexmedetomidine; IQR = interquartile range; SICU = Surgical Intensive Care Unit.

**Table 3:** Exploratory Outomes in Matched Cohort.

Variable	DXM-Propofol	Propofol	*p*-Value *^a^*	Median Difference (IQR)
	Median (IQR) or N (%)			
Duration of Propofol Infusion (hours)	96.0 (97.0)	118.6 (99.6)	0.07	–9.1 (120)
Duration of DXM Infusion (hours)	48.0 (65.0)			
Dexmedetomidine Dose (mcg/kg/hour)	0.32 (0.37)			
Propofol Dose (mcg/kg/min)	14.08 (14.4)	11.03 (10.6)	0.002	3.05 (4.2)
Fentanyl Dose (mcg/hour)	77.6 (71.6)	52.5 (48)	0.002	30.3 (93.7)
Percentage at Target Sedation (Riker = 4) While on Sedative Medication	34 (34)	36 (47)	0.32	–2 (13)
Percentage Above Target Sedation (Riker > 4) While on Sedative Medication	25 (27)	2 (10)	<0.001	23 (17)
Percentage Below Target Sedation (Riker < 4) While on Sedative Medication	37 (29)	52 (48)	<0.001	–12 (19)

DXM = dexmedetomidine; IQR = interquartile range; SICU = Surgical Intensive Care Unit; *^a^* Wilcoxon Rank Sum Test.

**Table 4:** Exploratory categorical variable results for matched cohorts by treatment group.

Variable	DXM-Propofol N (%)	Propofol N (%)	*p*-Value
Tracheostomy			
Yes	17 (11.9)	20 (13.9)	0.60 ^a^
No	126 (88.1)	123 (86.0)	
Fentanyl continuous infusion			
Yes	142 (99.3)	138 (96.5)	0.21 ^b^
No	1 (0.7)	5 (3.5)	
Continuous infusion neuromuscular blocking agent			
Yes	11 (7.7)	15 (10.5)	0.41 ^a^
No	132 (92.3)	128 (89.5)	
Triglyceride level			
≥200 mg/L	5 (3.5)	1 (0.7)	0.21 ^b^
<200 mg/L	138 (96.5)	142 (99.3)	
Use of any intermittent antipsychotic medication			
Yes	98 (68.5)	75 (52.4)	0.005 ^a^
No	45 (31.4)	68 (47.5)	
Use of any intermittent benzodiazepine medication			
Yes	52 (36.3)	50 (34.9)	0.81 ^a^
No	91 (63.6)	93 (65.0)	
Mean arterial blood pressure < 60 mm Hg			
Yes	109 (76.2)	77 (53.8)	< 0.001 ^a^
No	34 (23.8)	66 (46.1)	
Heart rate < 50 beats/minute			
Yes	8 (5.6)	28 (19.6)	< 0.001 ^a^
No	135 (94.4)	115 (80.4)	

^a^ Chi-Squared Test; ^b^ Fisher’s Exact Test; DXM = dexmedetomidine.

**Table 5:** Delivery of concomitant medication use in matched cohort by treatment group.

Medication	DXM-Propofol (*n* = 143) N (%)	Propofol (*n* = 143) N (%)	*p*-Value
Haloperidol	96 (67.1)	65 (45.5)	< 0.001 ^a^
Olanzapine	7 (4.90)	4 (2.8)	0.36 ^a^
Quetiapine	54 (37.7)	28 (19.6)	< 0.001 ^a^
Risperidone	0 (100)	0 (100)	NA
Fentanyl intermittent	58 (40.6)	74 (51.8)	0.06 ^a^
Hydromorphone drip	3 (2.1)	0	0.25 ^b^
Hydromorphone intermittent	58 (40.6)	53 (37.0)	0.54 ^a^
Morphine drip	0 (100)	0 (100)	NA
Morphine intermittent	25 (17.5)	29 (20.2)	0.55 ^a^
Oxycodone	105 (73.4)	95 (66.4)	0.20 ^a^
Hydrocodone	28 (19.6)	20 (13.9)	0.21 ^a^
Patient Controlled Analgesia	24 (16.8)	21 (14.7)	0.63 ^a^
Epidural	5 (3.5)	5 (3.5)	NA
Methadone	3 (2.1)	5 (3.5)	0.72 ^b^
Alprazolam	5 (3.5)	4 (2.8)	NA
Clonazepam	4 (2.8)	2 (1.4)	0.68 ^b^
Diazepam	6 (4.2)	3 (2.1)	0.50 ^b^
Lorazepam	30 (20.9)	20 (13.4)	0.12 ^a^
Midazolam	21 (14.7)	27 (18.9)	0.34 ^a^
Temazepam	2 (1.4)	0 (0)	0.50 ^b^

**^a^** Chi-Squared Test; ^b^ Fisher’s Exact Test; DXM = dexmedetomidine.

**Table 6:** Adjunctive dexmedetomidine studies.

First Author	Year	n	Control Group	Primary or Secondary Outcomes
Venn ^8^	2003	12	No	Efficacy of sedation
Shehabi ^10^	2004	20	No	Sedative and cardiovascular effects
Siobal ^9^	2006	5	No	Facilitate withdrawal of mechanical ventilation
Dasta ^17^	2006	9996 vs. 356	Yes	Hospital mortality, total hospital LOS, charges, # receiving mechanical ventilation, ventilation duration, ICU LOS
MacLaren ^11^	2007	40	No	Discontinuation or dosage reduction of other sedatives or fentanyl from the hour before to 6 h after starting DXM
Arpino ^12^	2008	20	No	Rate of extubation at 24 & 48 h post-DXM, mean time to extubation after DXM, mean rate of propofol/midazolam/morphine infusion before & after DXM initiation, heart rate and mean arterial pressure
Shehabi ^13^	2010	28	No	Effect of DXM on agitation during weaning of mechanical ventilation in critically ill patients
Reade ^14^	2016	39 vs. 32	Yes	Ventilator-free hours in the 7 days following randomization during the incident ICU admission

DXM = dexmedetomidine; LOS = length of stay.

## Supplementary Materials

The following are available online at http://www.mdpi.com/2226-4787/6/3/93/s1, Summary of Definitions and Explanations, University of Utah SICU Propofol Protocol, University of Utah SICU Dexmedetomidine Protocol.
