# Supplementary material for: Outcomes When Using Adjunct Dexmedetomidine with Propofol Sedation in Mechanically Ventilated Surgical Intensive Care Patients
Source: Pharmacy (Basel). 2018 Aug 28;6(3):93. doi: 10.3390/pharmacy6030093 (PMC6164835; doi:10.3390/pharmacy6030093)
Supplement: Supplementary file 1 [file pharmacy-06-00093-s001.pdf]

SUPPLEMENTAL CONTENT

Appendix: Outcomes when Using Adjunct Dexmedetomidine with Propofol Sedation in Mechanically Ventilated Surgical Intensive Care Patients

Table of Contents

Methods

Summary of Definitions and Explanations

Protocol 1 University of Utah SICU Propofol Protocol

Protocol 2 University of Utah SICU Dexmedetomidine Protocol

Summary of Definitions and Explanations from propensity score

APACHE II: calculated within the first 24 hours of ICU admission.

Bradycardia: (heart rate [HR] <50 beats)

Hypotension: (mean arterial pressure [MAP] <60 mmHg)

Hemodynamic instability: the treatment with an infusion of a vasopressor or inotrope, defined as: norepinephrine, epinephrine, phenylephrine, dopamine, vasopressin, dobutamine, or milrinone. Any of these agents required documented use as being given on any of the following days relative to intubation: one day before; the day of; or the day after intubation.

COPD: Defined per International Classification of Disease (ICD-9); diagnoses codes included codes beginning with: 490, 491, 492, 494, 496.

Study Exclusions by Admission Diagnoses: per International Classification of Disease (ICD-Version 9): Diagnosis codes related to severe head injuries and/or cervical spine fractures with cord involvement were excluded from the study population. In the ICD-9 index severe head injuries are specified as intracranial injuries with prolonged (more than 24 hours) loss of consciousness without the possibility of return to the pre-existing conscious level.

Excluded codes included the following:

800.5, 800.15, 800.25, 800.35, 800.45, 800.55, 800.65, 800.75, 800.85, 800.95, 801.05, 801.15, 801.25, 801.35, 801.45, 801.55, 801.65, 801.75, 801.85, 801.95, 803.05, 803.15, 803.25, 803.35, 803.45, 803.55, 803.65, 803.75, 803.85, 803.95, 850.4, 851.05, 851.15, 851.25, 851.35, 851.45, 851.55, 851.65, 851.75,

851.85, 851.95, 852.05, 852.15, 852.25, 852.35, 852.45, 852.55, 853.05, 853.15, 854.05, 854.15, 806.00, 806.01, 806.02, 806.03, 806.04, 806.05, 806.06, 806.07, 806.08, 806.09, 806.10, 806.11, 806.12, 806.13, 806.14, 806.15, 806.16, 806.17, 806.18, 806.19.

Admitting Service: There are a total of 17 admitting services. The table below shows the admitting service and the distribution of admitting services used in the propensity analysis. Services were merged into major service groupings based on the individual low total volume of cases within and compared to other services.

**Admitting Service within SICU**	**N**	**Admitting Services within Propensity Matching**
Bone Marrow Transplant	0	Bone Marrow Transplant
Cardiac Surgery	123	Cardio-Thoracic
Otolaryngology	7	Other
General Surgery	59	General Surgery
OB-GYN	3	Other
Orthopedics	6	Other
Other	1	Other
Thoracic Surgery	10	Cardio-Thoracic
Transplant Cardiac	4	Cardio-Thoracic
Transplant Liver	2	Transplant
Transplant Lung	0	Cardio-Thoracic
Transplant Pancreas + Kidney	0	Transplant
Transplant Pancreas	1	Transplant
Transplant Kidney	0	Transplant
Trauma	57	Trauma
Urology	6	Other
Vascular	7	Vascular

Time Period: To account for potential changes in critical care personnel, guidelines and institution protocols, a total of 8 time periods were used.

**Period**	**Dates**
1	April 2010 – September 2010
2	October 2010 – March 2011
3	April 2011 – September 2011
4	October 2011 – March 2012
5	April 2012 – September 2012
6	October 2012 – March 2013
7	April 2013 – September 2013
8	October 2013 – May 2014

Summary of Definitions and Explanations from results

Mechanical ventilation duration: first qualifying mechanical ventilation event based on duration and sedation use in patient SICU admission, subsequent mechanical ventilation durations were not included

Successful extubation: not reintubated within 24 hours

SICU length of stay: calculated as beginning with the date and time of SICU admission through the date and time of SICU discharge

SICU mortality: death during SICU admission

Confusion Assessment Method (CAM)-ICU: documented as positive, negative, or unable to assess (UTA) using one documentation per day method with a bias toward: positive, then negative, then UTA

Riker Sedation-Agitation Scale (SAS): documented on scale from 1-7 with target Riker score of 4 for all patients (excluding patients receiving continuous infusion neuromuscular blocking agents)

Concomitant medications: medications given in the SICU and either administered while a patient was receiving continuous infusion sedative medication (dexmedetomidine and/or propofol) or after sedation medication was discontinued

Paralysis: patient receiving continuous infusion neuromuscular blocking agent (cisatracurium or vecuronium) while mechanically ventilated

Triglyceride level: triglyceride level above 200 mg/dL while receiving continuous infusion sedative medication (dexmedetomidine and/or propofol)

Any antipsychotic medication: includes intermittent or scheduled doses of any of the following: haloperidol, olanzapine, quetiapine, or risperidone

Any benzodiazepine agent: includes intermittent as needed doses of any of the following: alprazolam, clonazepam, diazepam, lorazepam, midazolam, or temazepam

Protocol 1. University of Utah SICU Propofol Protocol

Titrate to Riker

General Sedation Path:

If comfort pain goal met and Riker > 4 start Propofol Drip.

Start Propofol drip at 10 mcg/kg/min if fentanyl drip rate is > 200 mcg/hr and Riker is > 4 with anticipated intubation time > 4 hours.

For Riker > 4: Increase rate by 10 mcg/kg/min every 10 minutes to Riker of 4

For Riker = 4: No Change

For Riker < 4: Decrease rate by 10 mcg/kg/min every 10 minutes to Riker of 4

Maximum Rate = 75 mcg/kg/min.

Protocol 2. University of Utah SICU Dexmedetomidine Protocol

All dosing based on actual (dosing) body weight. If patient is >135 kg, use 135 kg as dosing weight.

1. Give 0.5 mcg/kg load over 30 minutes (optional)

2. After load, start infusion at 0.5 mcg/kg/hour

3. If after 2 hours Riker > 4, increase infusion by 0.125 mcg/kg/hour

4. If necessary, repeat step 3 every 2 hours (Maximum dose is 1.5 mcg/kg/hour)

5. If at any time Riker < 4, decrease dexmedetomidine rate by 0.25 mcg/kg/hour

Guidelines for Titrating Off Propofol When Starting Dexmedetomidine

1. Initiate dexmedetomidine infusion and maintain current propofol rate for 1 hour.

2. Decrease propofol infusion rate by 10 mcg/kg/min per hour starting at hour 2.

3. Continue to decrease rate by 10 mcg/kg/min per hour until propofol is off.

*If propofol rate is 5 to 10 mcg/kg/min, decrease rate in half for one hour, then discontinue.

**If propofol rate is less than 5 mcg/kg/min, discontinue propofol infusion.
